# Raising bilingual autistic children in the UK: at the intersection between neurological and language diversity

**DOI:** 10.3389/fpsyt.2023.1250199

**Published:** 2023-09-08

**Authors:** Bérengère Galadriel Digard, Ellie Johnson, Draško Kašćelan, Rachael Davis

**Affiliations:** ^1^School of Philosophy, Psychology and Language Sciences, The University of Edinburgh, Edinburgh, United Kingdom; ^2^School of Health and Social Care, University of Essex, Colchester, United Kingdom; ^3^Division of Psychology, Sociology and Education, Queen Margaret University, Musselburgh, United Kingdom

**Keywords:** autism, bilingual, multilingual, neurodiversity, lived experience, family functioning, cultural minority, support

## Abstract

**Introduction:**

While research shows no negative effects of bilingualism on autistic children’s development, due to misconceptions around both autism and bilingualism, bilingual parents and educational/clinical practitioners who advise them often express unfounded concerns that exposing autistic children to more than one language will cause confusion and developmental delays. To understand the reasons that drive these misconceptions, this study focuses on: identifying factors that impact family decisions about (not) raising autistic children bilingually; attitudes toward bilingualism expressed by the community, doctors, family members, and teachers; sources of information about bilingualism and autism available to families.

**Methods:**

Through a mixed-method online survey, we explored these questions in 31 UK-based bilingual families with 34 autistic children (age *M* = 10.6 years; SD = 7.1).

**Results:**

The families reported choosing bilingualism for their autistic child primarily so that the child can communicate with family and community members. Attitudes toward bilingualism in their networks were predominantly positive, with a large portion of individuals not having opinions possibly due to lack of information. Only about 1/3 of parents had access to information on bilingualism and autism, mostly found on the internet.

**Discussion:**

We discuss these findings and offer future directions for research, practice, and battling stigmas around bilingualism and autism.

## Introduction

At least half the world’s population is estimated to be bilingual or live in a bilingual environment [(1), pp. 27–39], and logically, so should be half of the world’s autistic population. While attitudes towards bilingualism in neurotypical individuals are generally positive (with variation across and within communities), attitudes are less positive when it comes to autistic people (2). Autism is characterised by specific patterns of social communication and interaction, and repetitive behaviour and/or restricted interests. These characteristics are commonly seen through a deficit, disability or impairment lens in comparison to neurotypical norms and expectations, resulting in stereotypes and stigmatisation of autism as a condition.

Some of the wrongly founded assumptions about autism include the idea that exposing autistic children to more than one language causes confusion or negatively affects cognitive development. This has been observed in professional practice among educators and clinicians, who likely with a good intent but due to the lack of information and/or the presence of stigma, tend to advise bilingual families with autistic children to raise their children monolingually [e.g., see Yu (3)]. Early work by Kay-Raining Bird et al. (4), which explored this topic, and which predominantly included respondents from the United States and Canada, found that bilingualism was often not recommended/favoured by practitioners when advising bilingual families about raising their autistic children. This has been replicated in several other studies predominantly in the Canadian context (2). The UK context, however, remains rather underexplored in this respect with exception of a few recent studies. Specifically, among 11 families of autistic children in England and 5 families in Wales, Howard et al. (5) found predominantly negative or lack of advice by practitioners or other family members regarding bilingualism. Furthermore, in their work with seven practitioners in England and six in Wales, Howard et al. (6) identified some concerns about bilingualism not being feasible for all autistic children, as well as concerns that bilingualism might hinder a pupil’s literacy development and that language mixing might be cognitively demanding for an autistic child. As a result of these negative views regarding bilingualism in autism, many autistic children from bilingual families are denied access to learning their home language, and in turn, do not have the same opportunities to develop their cultural and linguistic identity as neurotypical bilingual children. Furthermore, since using the home language is essential for the participation of an autistic child in the family social life (7), the consequences of imposed monolingualism on an autistic child could have detrimental effects on communication and inclusion within a bilingual family or in fact community. Misconceptions about bilingualism and autism also play a role in a school context, where autistic pupils who need adjustments are frequently advised to drop language classes (8).

Contrary to concerns about exposing autistic children to more than one language, the growing body of evidence focusing on autism and bilingualism suggests that there are no negative effects of bilingualism on language or cognitive development [e.g., (9, 10)]. Research also suggests some tentative positive effects on cognition including facets of executive function skills [e.g., (11–13)] and crucially, in lived experiences. Specifically, parents describe increased familial connections and closeness when they are able to express themselves in their first language [e.g., (3, 14)] and autistic bilingual adults have expressed the benefits of bilingualism on domains such as widening access to work and social opportunities, social interactions, and self-understanding (15, 16).

The evidence of no harmful effects of bilingualism on development of autistic children suggests that the advice by practitioners not to expose autistic children to more than one language seems to be unfounded. Considering that there is a significant proportion of bilingual children in the UK [over 1.7 million pupils, equating to around 20% of school pupils (17)], as well as an estimate of more than 700,000 autistic individuals (18), it is crucial to better understand the views, attitudes and decision-making processes of parents regarding the linguistic environment for their autistic children. In the process, it is also vital to identify ongoing stigmas that might be present at the intersection of autistic and bilingual experiences. By using a questionnaire-based approach, comparable to Kay-Raining Bird et al. (4), and by expanding on the England- and Wales-based work of Howard et al. (5, 6) through the inclusion of participants from around the UK, the current research addresses the following questions:

1. What factors impact the decision-making processes around bilingual upbringing in UK-based bilingual families of autistic children?
2. What attitudes towards bilingualism do these families encounter among four main stakeholder networks (local community, doctors, family, and teachers)?
3. Where, if at all, do these families of autistic children find information about bilingualism and autism?

## Materials and methods

### Design and survey

The team developed an online, mixed-methods survey for UK-based bilingual parents of at least one child with a neurodevelopmental condition. The study received ethical approval from the School of Philosophy, Psychology and Language Sciences Research Ethics Committee from the University of Edinburgh, ethics approval number 22-2122.

### Measures

The online survey was designed by the authors due to the lack of an existing tool to address the needs posed by the broader research project (on bilingual families, neurodevelopmental conditions, and COVID-19 effects on family language practices), which this study focusing specifically on bilingualism and autism was a part of. In designing the survey, the authors relied on their previous experiences of investigating bilingualism and neurodiversity. Furthermore, one of the authors recently participated in an international project which included a design of an online tool to document bilingualism in children [see (19)]. The survey included the following: (1) a section about parents’ demographics, (2) a section about the first-born child including questions on their diagnoses (if any) and bilingualism (languages, exposure, and proficiency), (3) optional identical sections for up to seven other children if applicable, (4) a section about languages used with the child/ren, attitudes towards and the decision-making process concerning bilingualism, as well as about access to information on raising neurodivergent children bilingually, (5) a section on language use before COVID-19, (6) a section on language use at home during COVID-19, and (7) a section for any other comments. In this article we focus on data from all sections apart from those listed under (5) and (6). The survey is available on OSF at https://osf.io/gndb2/.

### Procedure

Participants were recruited by circulating the study details and flyers via social media, UK-based charities related to neurodevelopmental conditions (e.g., autism, ADHD, dyslexia, and learning disabilities), as well as personal and professional contacts. As bilingual families with autistic (and neurodivergent) children are hard to recruit in the UK, it was decided that the best approach to obtain responses was to use every possible network through which prospective participants could be identified and recruited.

The online survey was hosted on a GDPR (General Data Protection Regulations)-compliant platform^1^ and was active from December 2021 to June 2023. Respondents accessed the study via a general link to the online survey, which started with a participant information sheet and a consent form. Respondents were provided with the research team’s contact details and were able to access the survey after providing informed consent. They were also informed that most questions were not mandatory, and respondents could choose not to answer them. Respondents were told that they could go back, or pause the survey, and that it would take between 10 and 30 min to complete depending on the number of children that they have in their family. Respondents were also informed that they had 2 weeks to contact the research team to withdraw their data from the study.

### Analysis

The descriptive analysis of the quantitative data was conducted in R (version 3.5.3). Responses to the open-ended questions were used to qualitatively supplement quantitative data. All data was inspected both in R and in Excel.

## Results

### Participants

A total of 40 parents completed the survey, 31 of whom were parents of at least one autistic child. This research focuses on this sample of 31 respondents (mean age = 44.7 years, SD = 7.3, range = 32–69; 26 females, 4 males, 1 not disclosed) who together represented 62 children including a total of 34 with a clinical diagnosis of autism. Amongst the parents, two respondents chose to disclose a diagnosis of autism, and two a diagnosis of ADHD (mean age at diagnosis = 24.7 years, SD = 17.2, range = 5–37), while two self-identified as autistic. Some respondents also chose to disclose their partner’s neurodevelopmental diagnoses: autism (2), ADHD (2), dyslexia (1), and learning disability (1). Respondents reported a total of 22 different first languages (Arabic, Bengali, Cantonese, Czech, Dutch, English, Finnish, German, Greek, Hebrew, Irish/Gaeilge, Italian, Japanese, Polish, Portuguese, Romanian, Russian, Scots, Slovenian, Spanish, Turkish, and Twi), and 21 different countries of birth (Bangladesh, Brazil, Czech Republic, Finland, Germany, Ghana, Greece, Hong Kong, Israel, Italy, Japan, Moldova, Netherlands, Poland, Russia, Saudi Arabia, Slovenia, Spain, Turkey, United Kingdom, and Venezuela).

Of the 34 autistic children represented (mean age = 10.6 years, SD = 7.1, range = 3–38; 7 females, 26 males, 1 non-binary), 21 only had a clinical diagnosis of autism, while 13 had both autism and one or more other clinical diagnoses: ADHD (5), developmental language disorders (6), dyslexia (1), dyspraxia (1), learning disability (5), or other neurodevelopmental conditions (4). Mean age at their first referral for their first neurodevelopmental assessment was 5.1 years (SD = 5.4, range = 1–30), and mean age when receiving their first clinical diagnosis was 6.4 (SD = 5.4, range = 2–30).

### Language profiles of the children

**FIGURE 1:**
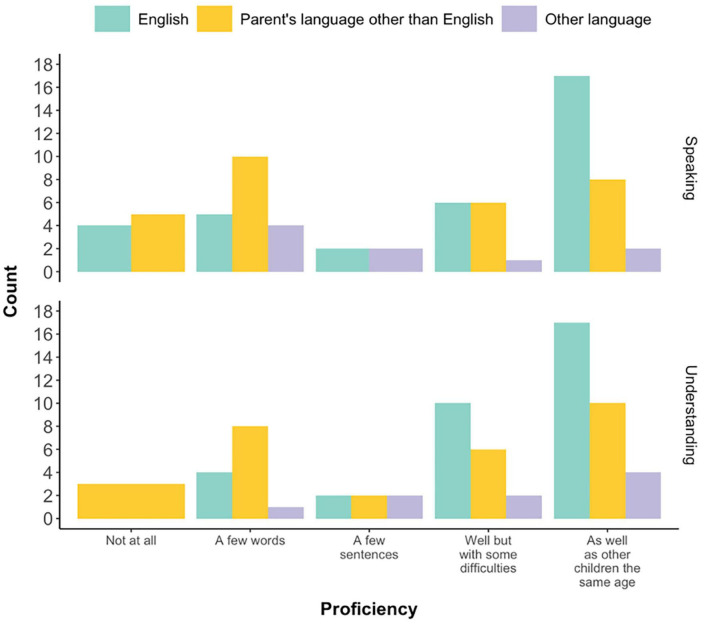
Parent-reported oral expression and oral comprehension skills of their autistic children in the languages they are exposed to. For each child, the proficiency scores were not necessarily provided for all three groups of languages presented in the figure: English (green), responding parent’s language other than English (yellow), and other language that the child is exposed to (purple). This means that the proficiency of some children was rated for English and parent’s language other than English only, while for some other children this included English and other language only. There were also trilingual children, for whom the rating was given for all three languages.

Figure 1 shows the respondents’ rating of their autistic children’s oral expression and comprehension skills in: (a) English (green), (b) responding^2^ parent’s first (or main) language other than English (yellow), and (c) another language that the child is exposed to, which is either a language that they acquired from their other parent and/or from other interlocutors/contexts (purple). All 34 children had proficiency skills rated in English,^3^ 29 had their language proficiency reported for the responding parent’s first language other than English, and 9 children had proficiency reported in another language. About 50% of the children (*n* = 17) had English understanding and speaking skills similar to that of their same-aged peers, while some children had a few sentences or less in their comprehension (18%, *n* = 6) and expression (29%, *n* = 10) skills. Overall language skills were lower in their main non-English language (i.e., the parent’s first language) compared to English, with 34% (*n* = 10) having similar comprehension skills as their peers, and 28% (*n* = 8) for expressive skills. However, many children had language skills of a few sentences or less in both comprehension (45%, *n* = 13) and expression (52%, *n* = 15). When it comes to children speaking an additional language (*n* = 9), 44% (*n* = 4) had comprehension skills similar to that of their same-aged peers, though most children (67%, *n* = 6) were reported to have expressive skills of a few sentences or less.

### Decisions on bilingual upbringing

#### Quantitative responses

**FIGURE 2:**
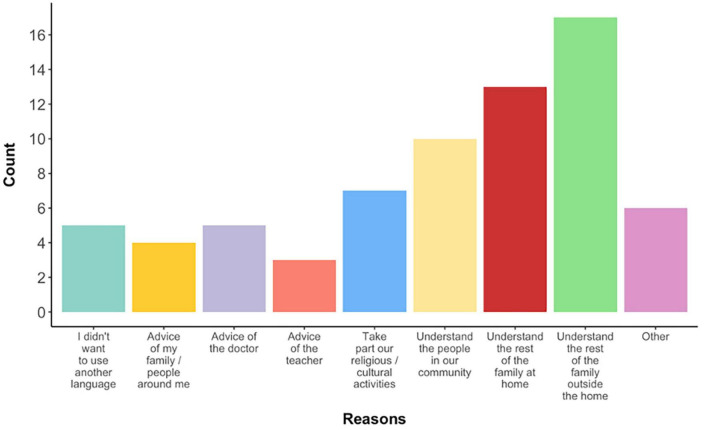
Respondents’ reasons (in numbers) for choosing bilingual upbringing for their autistic children.

Of the 31 respondents, 27 reported their children were raised with more than one language at home, 26 reported that before their child’s diagnosis they were planning to raise them bilingually, and 12 reported that receiving the diagnosis had led them to reconsider their decision. When asked to select the reasons that led them to decide what languages they would use with their child (Figure 2), most of these 27 respondents listed as a reason to choose bilingualism a wish for their child to understand their family at home (48%, *n* = 13), their extended family outside the home (63%, *n* = 17), and people in their community (37%, *n* = 10).

#### Qualitative responses

To supplement the counts presented in Figure 2, we looked at the open-ended questions regarding decisions on bilingual upbringing. Considering that the open-ended questions were optional and not frequent, we did not collect a number of qualitative responses deemed large enough to require a comprehensive coding approach. We therefore grouped and addressed these open-ended responses by looking at three cohorts of parents that emerged from our data based on the families’ bilingual (or monolingual) practices. These included: (1) parents who do not speak more than one language with their child/ren, (2) parents who indicated that they speak more than one language to their child/ren and who *intended* to use more than one language with their autistic child/ren prior to their child/ren’s diagnosis, and (3) parents who indicated that they speak more than one language to their child/ren and who *did not intend* to use multiple languages with their autistic child/ren prior to their child/ren’s diagnosis.

The first focus was on responses from the four participants who do not speak more than one language with their child/ren. While three participants initially intended to use more than one language with their child/ren, they changed their minds upon their child receiving a diagnosis. The following clarifications were provided by two families:

Parent 27: “My children 1st language is English although my husband and I speak French. My 1st born was exposed to our native languages, and we spoke to him in French. However, when we realised he had a language delay and was referred for an autism diagnosis, we decided to use English with him to help him ‘catch up’ on the use of English language.” They further added that they “gave up on using our native languages in favour of English to help them develop language and communication skills used in the community they live in.”^4^
Parent 37: “Basically, our view is about practicalities. We live in an English-speaking country, and we decided that we should concentrate in it to make sure our children are able to speak the language in the country they live in. Besides, my wife and her family are native English speakers.”

The second focus of responses included participants who indicated that they use more than one language with their child/ren and who intended to use more than one language with their autistic child/ren *prior* to their child/ren’s diagnosis. From the 23 parents in this sample, six respondents changed the way in which they use their languages with their autistic child/ren after they received a diagnosis. These six parents provided the following clarifications:

Parent 2: “Were advised to speak only Arabic with him. Then he joined English language nursery here at [anonymised]. We started to use more English, as we thought he might prefer English. I attended a seminar by [anonymised] and decided to speak both with him.” This parent also clarified that “The father’s Ph.D. topic has a minor in family language policy. I have decent information on how to raise my child bilingually. Yet I have to admit it didn’t help much with my neurodivergent child.”
Parent 4: “Speech and Language therapist suggested our son with special needs couldn’t learn the language as others so we should stick with one language with him.”
Parent 10: “We felt that there wasn’t clear evidence from the speech and language therapist that using both languages at home was beneficial or on the other hand confusing for our son as already there was speech delay, and it is still the case.”
Parent 26: “At the beginning she wasn’t speak any word and start to speak and language therapy in English. Unfortunately, I have to pick the English over the my mother tongue.”
Parent 35: “It’s hard to know because of my child’s language delay how much he understands. I have read leaflets that hearing two languages won’t confuse him, but to me it already seems that English alone confuses him. His speech therapist did not know much about bilingualism and had no advice. (He has now been discharged anyway).”
Parent 40: “Because the younger child is non-verbal, our communication has to be very simple. Another language is used sometimes is purely out of coincidence or that had been used since birth, which is also at a minimal level due to his understanding of the spoken word. For the teenager, I changed my mind about pushing him to learn my mother tongue because of his autism. I didn’t want to give him extra pressure but my husband and I use Cantonese and French on some occasions for fun or for special greetings. This is so that our son can grow up knowing the existence of these languages esp Cantonese but he doesn’t necessarily have to learn it, as long as he doesn’t resent it is our aim. He loves French at school and has been learning Spanish, too. My own experience of living in [anonymised] has perhaps had an influence on him.”

In the same cohort of 23 families, 17 decided to continue using multiple languages with their child/ren *after* a diagnosis was received. In addition to reasons summarised in Figure 2, several participants provided clarifications:

Parent 8: “I thought it could be an opportunity to have a job as a translator/interpreter if they want. It could be a backup job while they are looking for a their dream job or a source of additional income if needed.”
Parent 17: “Natural to speak whichever language best conveys the emotions and communications you aim to convey. i.e., what feels right.”
Parent 31: “Support their general development of language, and awareness that not everyone speaks English in the world.”
Parent 34: “The change to learn my language without accent and the fathers language without accent.”
Parent 36: “As a speakers of a minority language it was really important for me for reasons of emotion and identity and survival of our language too as well as all the above reasons. The children’s father and I had both been brought up bilingually and had very positive experiences of this (and the support of our families).”

The final subset of responses included four parents who reported currently using more than one language with their child/ren but who did not intend to use multiple languages with their autistic child/ren *before* the diagnosis was received. Of these four families, three changed their mind after their child received a diagnosis, and one did not. From three parents who indicated changing the way in which they speak with their autistic child/ren after the diagnosis, one ended up opting for using English only while two parents opted in favour of bilingualism. Those two parents indicated the following:

Parent 6: “We adjusted our communication in both languages. We made our sentences short and with clear instructions. Also we spoke slowly. In addition, we started speaking English to our children at home when doing homework or speech and language therapist therapies, and also in the public we tend to speak English more than Russian to share the common language environment and to include other people in our conversations with children.”
Parent 18: “I became more understanding of my child’s needs.”

Looking at the combination of quantitative (Figure 2) and qualitative responses, the reason for (not) choosing to raise autistic children bilingually varied widely. The findings are addressed in detail in section “Discussion.”

### Attitudes towards bilingualism

**FIGURE 3:**
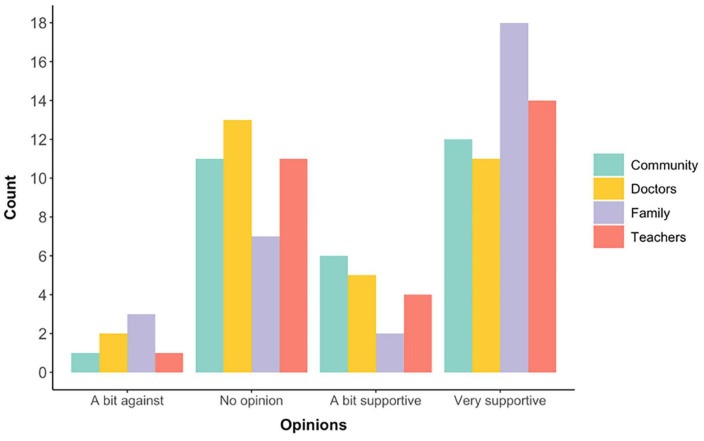
Attitudes towards a bilingual upbringing (in numbers) for autistic children reported for four cohorts (community members, clinicians, family members, and teachers).

Respondents reported a similar pattern of attitudes from community members, clinicians, family members, and teachers, towards bilingual upbringing for autistic children (Figure 3). No respondents reported very unsupportive attitudes from either group, with most common attitudes being no opinion or a very supportive attitude across all groups of key partners. An overall positive (“a bit” or “very” supportive) attitude towards a bilingual upbringing for autistic children was reported from community members (18 respondents, 58%), clinicians (16 respondents, 52%), family relatives (20 respondents, 65%), and educators (18 respondents, 58%).

### Sources of information regarding bilingualism

**FIGURE 4:**
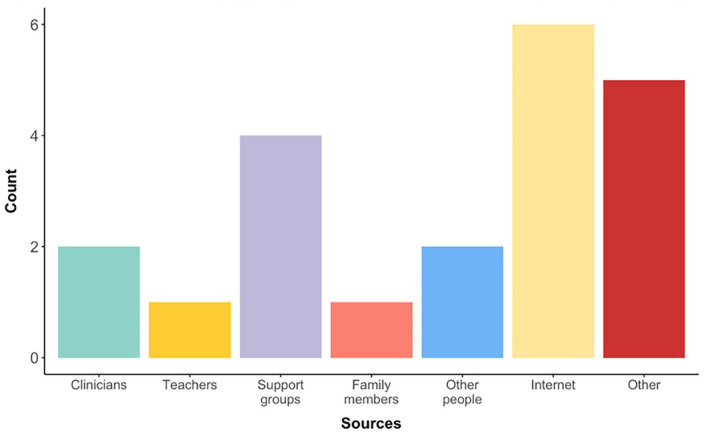
Sources of information regarding bilingual upbringing for autistic children. Selecting “Other” allowed for open entries, which were completed by respondents with “Books,” “Speech and Language Therapists” (which could have been placed under “Clinicians”), “Webinar,” and “Own research/publications.”

Ten respondents (32%) received information about raising autistic children in a bilingual context (Figure 4). The majority of participants had found information online (*n* = 6), or through support groups (*n* = 4). Few reported receiving information from their clinicians (*n* = 2) or teachers (*n* = 1). Of these 10 respondents, 5 indicated being aware of the research in this field.

## Discussion

This study aimed to elicit the views of UK-based parents of autistic children who have the potential to be raised in a bilingual environment. In this section we first discuss three questions which were the focus of our study: the reasons why parents decided (not) to raise their child/ren in a bilingual environment, the sources of information that affected such decisions, and the influence of attitudes from various groups on the parental decision-making process. Following this, we summarise a desiderata for improved practice and discuss the feasibility of achieving these targets in the UK socio-political context. We also comment on the importance of moving away from the exceptionalism of bilingualism in order to prevent reinforcing myths/stigmas about both bilingualism and autism. We acknowledge the limitations of our study before closing with concluding remarks.

### Reasons for (not) choosing bilingualism

The most common reason that parents listed for choosing a bilingual environment for their children was to provide additional opportunities for their children to better understand the family both inside and outside the home and to integrate with members of their community. This data demonstrates a somewhat obvious fact that bilingualism is likely a requirement for functional relationships within a local community and in family circles where more than one language is used. Consequently, advising against bilingualism can lead to a serious damage to family/community dynamics and wellbeing [for discussions on the importance of bilingualism for child wellbeing in general, see work by Müller et al. (20)].

As parents in the survey were asked this question in relation to all their children (including neurotypical children), it is likely that in some of these families who generally use multiple languages, they raised their autistic children monolingually for a variety of reasons. The questions regarding the influence of a diagnosis on parents’ decisions to raise their children bilingually/monolingually helped to disentangle this. Six families indicated that they changed their child’s linguistic environment following the diagnosis, although some did return to bilingualism – highlighting the dynamic nature of this decision-making process.

Open-ended responses further revealed several factors that affect parental decisions on bilingualism. These included advice from speech and language therapists (SLTs), situations in which SLTs did not feel confident in providing advice, or where access to language therapy was only available in the societal language. Parents also described their own concerns that hearing or speaking more than one language could be confusing, and that for minimally verbal children, bilingual environments could cause additional pressure on the child using language in the future. Importantly, one of the respondents who returned to bilingualism with their autistic child reported that even though their partner knew about bilingualism from their own doctoral research, this knowledge did not help when raising their neurodivergent child. This point is addressed further below in the discussion.

This qualitative dataset of six families supplements the quantitative data presented. Although this is a small sample, the testimonies are in line with reflections from previous research from families with autistic children [e.g., (3–5)]. To understand the prominence of these concerns and to map the dynamic nature of decision-making about bilingual upbringing, a follow-up qualitative approach using semi-structured interviews could offer a more comprehensive understanding of the question which we focused on in this study [as per Howard et al. (5), for example].

### Information available to parents and relevant stakeholders

As our data indicated that the advice about bilingualism somewhat affected parental decisions on raising their autistic children with(out) multiple languages, we now focus on the research question inquiring about available sources of information on bilingualism and autism. Only 10 respondents reported receiving information about raising their children bilingually. Even within this small cohort, the sources of information varied extensively, with the majority of individuals finding information online. This sets a significant task ahead when it comes to communicating scientific findings beyond academia.

What can be taken from the current findings is the following list of actions which would contribute to a more informed and accessible bilingual upbringing of autistic children: (1) a need to provide training for SLTs about the intersection of bilingualism and autism, and (2) a need to communicate relevant research to other key stakeholders (e.g., teachers, parents, and policy makers) in an accessible format. Pathways to undertaking these actions are many. We briefly discuss these points in relation to some relevant practices from the UK context.

When it comes to the training of future SLTs, as highlighted in Pert [(21), p. 204], including teaching on bilingualism and cultural diversity in the curriculum of pre-qualification SLT programmes would contribute to a solution. Indeed, the Royal College of Speech and Language Therapists^5^ (RCSLT) curriculum guidance for the pre-registration education of SLTs contains bilingualism^6^ as a content area integral to the development of future SLT skills (22). Anecdotally, we are aware that the contact hours dedicated to bilingualism in UK SLT programmes vary extensively – while some programmes dedicate a whole module to this topic, others have the topic integrated in various courses. Moreover, the increase in integrating the content on bilingualism in the UK SLT programmes seems to have occurred in the last few years. Unfortunately, we lack comprehensive information on how systematic this is across the UK universities. These speculative conclusions based on personal communication imply that SLTs who were trained in the last few decades might not have received the same amount of training on working with bilingual children as the newer generations of SLTs have/will. While this could be somewhat mitigated through continuing professional development (CPD) training, the availability or free access to relevant courses is not always guaranteed. We highlight that our conclusions on this point are based on anecdotal evidence. More research in this area and investment in training SLTs is necessary in order to improve the work of SLTs with bilingual families.

Bilingual parents of autistic children, as well as other relevant stakeholders, such as teachers and policy makers, are also in dire need for accessible information on interactions between bilingualism and autism. In a recent initiative from the University of Edinburgh and Queen Margaret University, accessible resources on autism and bilingualism have been produced in written format (in multiple languages) as well as in the form of a video (23, 24). Increasing such engagements of researchers with the relevant stakeholders could contribute to providing accessible state-of-the-art information necessary to inform choices about bilingual upbringing in autism. What is missing, however, is more extensive research and communication about not only the interaction of bilingualism and autism but also on how to raise autistic children bilingually in the UK context. Going forward, including parents and autistic individuals in the study designs could help direct research on community priorities and ensure the facilitation of access to bilingual environments as well as to strategies for bilingual upbringing.

### Attitudes towards bilingualism

The study also explored the attitudes towards bilingualism and autism which parents of autistic children encountered among the local community, doctors, family members, and teachers. In the current sample, we identified a difference in attitudes towards bilingual upbringing in comparison to earlier work on this topic. For example, in an international study with most participants from Canada and the US, Kay-Raining Bird et al. (4) found that the majority of parents (regardless of whether they raised their autistic children monolingually or bilingually) received negative advice regarding bilingualism. More recently and in the UK context, Howard et al. (6) found that a group of practitioners expressed concerns about the feasibility of bilingualism for autistic children. Howard et al. (5) further confirmed this trend of negative advice regarding bilingualism and autism offered to parents by practitioners or family members. In the current sample, the majority of parents reported supportive attitudes regarding bilingualism from key stakeholder groups (local community, doctors, family members, and teachers). Importantly, while these groups had some instances of negative attitudes towards bilingualism, no group expressed strong negative views. The increase in positive attitudes in comparison to earlier literature on the topic could be partly due to the increase in the number of studies with autistic bilinguals over the last decade, and subsequently more research demonstrating a lack of negative effects of bilingualism. As Prévost and Tuller (10) report in their recent review of studies with autistic bilinguals, around half of the papers on the topic (11 out of 20) appeared in the last 3 years. We return to the discussion on the effect of bilingualism, particularly to the quest for bilingual advantage further below.

While the predominantly positive attitudes towards bilingualism in the current sample are welcome, we cannot claim with certainty that they can be generalised throughout the UK, especially bearing in mind our sample size. Additionally, to better understand any stigma associated with bilingualism, it is important to look beyond the specific research question that we focused on (i.e., attitudes towards bilingualism that bilingual families of autistic children encountered in their networks) and situate our research in the wider UK context. For instance, recent data from YouGov (25) indicated that about a quarter of 1,461 surveyed British participants expressed being bothered when they hear people speaking in a language other than English. Such a strong presence of xenophobic attitudes likely impacts views towards bilingualism across sectors, including in the stakeholder networks which we inquired about in the present study.

When it comes to the UK SLT practice in particular, Pert [(21), p. 198] notes that a negative attitude towards languages other than the mainstream ones (such as English or Welsh) is one of the factors likely related to predominantly English-only approaches to assessment and intervention. In interaction with other factors that come into play (e.g., the cost of interpreters in the SLT practice, the availability of resources in languages other than English, the working conditions of practitioners, etc.), this English-only SLT support can thus affect bilingual families’ decision-making process about maintaining the use of more than one language with their autistic children.

A striking finding from this study is that within each of the four stakeholder groups (i.e., local community, doctors, family members, and teachers), there is a significant portion of individuals with no opinions on bilingualism (35, 42, 23, and 35% respectively). For comparison, Howard et al. (5) identified that no advice regarding bilingualism was the most frequent answer in their UK-based cohort of parents. Despite the increase in studies with autistic bilinguals (as pointed out above) and positive attitudes towards bilingualism which we identified, the significant number of parents reporting no opinions received across their networks could indicate that there is somewhat still a lack of easily available information on bilingualism. This is particularly worrying when it comes to teachers and clinicians, and it implies a need for providing resources and comprehensive training on bilingual development to these stakeholder groups, as has been suggested by Davis et al. (26), Pert [(21), pp. 204–206], and in our discussion above.

While the attitudes towards bilingualism in our sample are predominantly positive, the reality of raising autistic children bilingually remains challenging. As indicated by a parent in the current sample, attending a session on bilingualism can help them make decisions about raising their children bilingually. Nevertheless, as the same parent pointed out, even with relevant information, parents and autistic children still face challenges when it comes to a bilingual upbringing. These concerns corroborate those identified in a group of practitioners and parents in Howard et al. (5, 6), where the feasibility of bilingualism for all autistic children was found to be questionable. In addition to working on changing negative attitudes on bilingualism, stronger support services, such as the investment of government funding in the National Healthcare Service (NHS), in the education sector, and in local councils is necessary to ensure access to bilingualism for more autistic children, while acknowledging that individualised advice from clinicians and educators will still have to be given on a case-by-case basis (6).

### Desiderata and challenging the status quo

The analysis of our dataset leaves us with the following desiderata for future practice: (1) a need for more comprehensive training/education of SLTs regarding bilingualism and autism, (2) a need to communicate research findings on autism and bilingualism to other relevant stakeholders in an accessible way (parents, teachers, doctors, and policy makers), (3) a need to confront stigmas about both autism and bilingualism and their intersection particularly in relation to negative attitudes and xenophobic views, (4) a need to support bilingual families with autistic children and provide them not only with information on the current research on the topic, but also with strategies and resources for bilingual upbringing. Achieving these targets requires an immense amount of work and collaboration by several groups of stakeholders, such as healthcare professionals, educators working in the primary, secondary and higher education context, policy makers, as well as families of children with autism. While our desiderata is a wish-list akin to suggestions from other work on the topic [e.g., (5, 6, 21, 27)], we find that it is important to address it by situating the findings of our research into the UK socio-political context in order to assess its feasibility.

First, we focus on working conditions of relevant stakeholder groups. A recent survey by RCSLT (28) indicated a national average vacancy rate in the SLT profession of 23%. The same report identified a need for improved workforce planning, a need to train more students, to retain and develop the workforce, as well as a need for improvement in funding. Whether this will change, ultimately depends on government support and funding given to both the health and the education services. In recent years, the austerity experienced across the UK resulted in industrial actions both in healthcare and education. The working conditions of teachers, health and support workers, as well as those in higher education who conduct research and provide training/teaching for future health and education workforce have significantly deteriorated. These conditions inevitably affect the quality of what is being investigated, communicated, taught and disseminated to stakeholders and families with autistic children. While our research-informed desiderata outlines necessities of the communities that we work with, solidarity and participation in the ongoing industrial actions as well as socio-political engagement are vital for systemic changes to take place – this is what will ultimately improve the quality of life of bilingual families with autistic children. Maintaining the *status quo* will not contribute to improving lives and breaking stigmas that both autistic and bilingual communities face, and particularly those at their intersection.

Moving away from the *status quo* will also be important to ensure changes in attitudes towards bilingualism and autism. Considering the presence of xenophobic views in the UK, maintaining the *status quo* as SLTs, researchers, teachers, and other clinicians makes us complicit with discrimination that affects bilingual families in general, as well as those with autistic children. A discussion on challenging *status quo* in relation to SLT practice in the bilingual context is offered in Pert [(21), pp. 198–215]. To those points, we add a need for participation in and solidarity with relevant industrial actions, as well as other forms of activism which will drive the long overdue systemic changes.

### Avoiding the exceptionalism of bilingualism

The final point which we address goes beyond the scope of this study, but it is particularly relevant when considering access to information on bilingualism and autism. As it has been discussed above, the internet was identified as the most common source of information on the topic in our sample. When it comes to bilingualism in particular, the plethora of information available online and directed at the general audience can sometimes overestimate the implications of bilingualism as an experience and reinforce myths or even stereotypes about using multiple languages. An extreme example of lay interpretations of research attempting to make bilingualism sound attractive can be found in articles such as that by Riotta (29).

These attempts to present bilingualism as an exceptional experience are somewhat existent in our own work and research culture. They can be particularly problematic when it comes to investigating bilingualism, autism, and cognition. As mentioned earlier, work with autistic bilinguals indicates potential benefits in cognitive skills [e.g., (12)]. On the one hand, exploring possible cognitive benefits of bilingualism could be seen as an attempt to encourage families to preserve the use of multiple languages with their autistic children. Indeed, this has been observed in Howard et al. (5). On the other hand, while these investigations are likely well-intended, approaching bilingualism exploration with reference to monolinguals and identifying possible cognitive advantages could be seen as another way of attempting to justify the value of bilingualism in a space dominated by monolingualism-centred expectations – as if the bilingual experience does not have its own value and is not worthy of exploration unless it can offer particular cognitive advantages. Furthermore, these approaches could backfire and have a detrimental effect on the family decision-making process regarding preserving bilingual practice. For instance, if a study identifies that autistic bilinguals are significantly slower on a particular executive function task than autistic monolinguals, which might be translated to being 200 ms slower, is it worth dropping one language for the fear that bilingual exposure might have something to do with this? Specifically, is the cost of 200 ms on one executive function task more important for the wellbeing of an autistic child and their family than the ability to communicate with the family and their network of speakers by using all languages commonly present in their environment? The majority of our sample indicated that they chose bilingual upbringing for their autistic children in order to enable them to communicate with the family and the members of the local community. The importance of this has also been noted in previous work [e.g., (5, 7)]. The shift in research focus is therefore required, as our pursuit for cognitive advantages of bilingualism in autism inevitably contributes to the exceptionalism of bilingual experience and can have negative effects on family decision-making process and ultimately wellbeing.

The bilingual cognitive advantage quest in autism can also (unwillingly) contribute to reinforcing stigmas about autism. For instance, if the common assumption is that bilingualism improves specific aspects of cognition, it might mitigate certain difficulties in autism – therefore, it is good for autistic individuals. Such an approach is inadequate as it assumes that the cognitive profile of autism automatically equates to deficits and the almighty bilingualism is there to save the day. Most of us are guilty of approaching research on bilingualism and autism in such a way. Instead, we should explore whether the existing ways in which we tap into cognitive skills (which are primarily designed with a neurotypical individual in mind), are affecting how autistic individuals perform on these measures. Moving away from problematic designs could help us focus on investigations of bilingual experience in autism that reduce the stigmas of both being autistic and being exposed to more than one language as an autistic individual. Going forward it is necessary to centre participatory approaches – through which lived experiences of autistic individuals can be incorporated into the exploration of bilingualism – thus minimising biases that we as neurotypical researchers bring to the field. Importantly, as researchers we need to prioritise communicating findings beyond academia in a responsible way, to prevent situations in which our advice could contribute to stigmatising autism, and in relation to bilingualism lead families to drop one of their languages when there is no reason strong enough to do so. For further criticism of the bilingual advantage quest beyond the work in autism, see Leivada et al. (30). Rothman et al. (31) also offer a recent discussion on monolingualism-centred investigations of bilinguals which are important to consider in future research.

### Limitations

One of the limitations of this study is what might be perceived as a small number of participants for broader generalisations. However, over half of the published studies on autism and bilingualism to date have included 20 or fewer autistic bilinguals (10). As autistic bilinguals represent a hard-to-recruit population in the UK, expectations of studies with larger samples are somewhat unrealistic without a longer period even for cross sectional data collection and collaborative efforts (10). We experienced this difficulty in the current study, as it took 18 months to collect responses from 40 families with children with neurodevelopmental conditions (including 31 bilingual families with 34 autistic children). Considering these circumstances, our sample is a significant contribution to the field of bilingual development in autistic individuals. As all respondents are based in one country, this also enables us to situate their experiences in a single context. We do note, however, that although larger samples can likely lead to more relevant conclusions for a larger proportion of the target population, significant variability in both bilingual and autistic experiences could be better captured with the addition of qualitative research. We have attempted to include this aspect in our survey by adding some open-ended questions, but offering opportunities for interviews or alternative modes of communication could improve future work in this area.

As the largest portion of our data collection period took place during the pandemic, an online survey was considered more practical rather than offering options for an in-person interview. The option of offering an online interview was considered, but as it would require asking for more commitment by the participant (e.g., finding adequate time for an online meeting that fits both the participant and the researcher), this option was not implemented. These restrictive conditions likely affected the number of recruited participants. Therefore, more inclusive participation approaches should be offered to prospective participants in future, not only to increase the participant numbers, but also to enable participants to choose a participation mode most suited to their needs.

A second limitation includes not compensating the respondents for their participation in the study. This was determined by limited funds available to the authors of the paper, some of whom were on precarious contracts during the time of the study design, data collection and analysis, and writing. On the one hand, as suggested by Pellicano et al. (32) in their recent discussion of data integrity from online studies on autism, the lack of financial incentive for participation may decrease the chances of recruiting any financially motivated study scammers. Nevertheless, the lack of participation reimbursement raises several issues, as pointed out in Pellicano et al. (32): a suggestion that participants’ time is not valued, exploitation of the autistic community for research purposes, damaging trust between researchers and autistic communities, exclusion of lower-income participants, and bias towards more financially comfortable people and those with intrinsic interest in the research topic. Apart from expressing gratitude to our participants, we need to do better in future research and ensure just compensation for participation time.

## Conclusion

This work expands on previous evidence regarding the decision-making process that bilingual families experience when raising autistic children. We provide evidence of increased positive attitudes towards bilingualism and autism in the UK context. Nevertheless, a significant portion of key stakeholder groups (clinicians, teachers, members of the family, and the local community) still lack accessible information on bilingualism and autism. We offer suggestions on how to move forward in this area of research and highlight the importance of abandoning the *status quo* across our professional sectors for systemic changes to take place. It is important to acknowledge that our work is situated in a Western-centric context and approach to thinking about bilingualism and autism. What is also necessary is the engagement with alternative approaches of how bilingualism is conceptualised and experienced, or rather engagement with individuals and communities whose views and experiences have previously been pathologized and pushed to the periphery [for relevant discussions in the educational and the clinical contexts, see García et al. (33) and Nair et al. (34)]. This change of perspectives will play a crucial role in reducing stigma around bilingualism, and ultimately about ensuring access to bilingualism for autistic people.

## Data availability statement

The raw data supporting the conclusions of this article will be made available by the authors, without undue reservation.

## Ethics statement

The studies involving humans were approved by the School of Philosophy, Psychology and Language Sciences Research Ethics Committee, University of Edinburgh. The studies were conducted in accordance with the local legislation and institutional requirements. The participants provided their written informed consent to participate in this study. Written informed consent was obtained from the individual(s) for the publication of any potentially identifiable images or data included in this article.

## Author contributions

BD, EJ, DK, and RD: study conceptualisation, study design, and data collection. BD, EJ, and DK: data analysis. BD, DK, and RD: manuscript writing. All authors contributed to the article and approved the submitted version.

## Footnotes

1
www.onlinesurveys.ac.uk

2 By responding parent we refer to the parent who completed the online survey.

3 The comprehension skills in English were not reported for one child. Therefore, we have English expressive skills scores for all 34 children, while there are English comprehension skills scores for 33 participants.

4 To preserve the authenticity of respondents’ voices, we did not correct their writing into standardised English. Slight modifications were made only when we removed certain details for anonymisation purposes, which we indicated in square brackets.

5 The Royal College of Speech and Language Therapists is the professional body for SLTs in the UK.

6 The RCSLT guidance uses the term multilingualism rather than bilingualism. As in this article we used the term bilingualism to refer to the knowledge/use of two or more languages, we chose to use the term bilingualism here for consistency purposes.
